# Association Between Consumption of Ultra‐Processed Foods and Adiposity Measures in Lactating Women

**DOI:** 10.1002/fsn3.70488

**Published:** 2025-06-27

**Authors:** Lara Virginia Pessoa de Lima, Lígia Rejane Siqueira Garcia, Juliana Morais de Sousa, Priscila Gomes de Oliveira, Nicolie Mattenhauer de Oliveira, Juliana Fernandes dos Santos Dametto, Danielle Soares Bezerra, Karla Danielly da Silva Ribeiro

**Affiliations:** ^1^ Postgraduate Program in Nutrition Federal University of Rio Grande do Norte Natal Rio Grande do Norte Brazil; ^2^ Health Sciences College of Trairi Federal University of Rio Grande do Norte Santa Cruz Rio Grande do Norte Brazil; ^3^ Nutrition Undergraduate Program Federal University of Rio Grande do Norte Natal Rio Grande do Norte Brazil; ^4^ Department of Nutrition Federal University of Rio Grande do Norte Natal Rio Grande do Norte Brazil

**Keywords:** breastfeeding, lactation, Nova classification, overweight, postpartum, weight retention

## Abstract

This study aimed to evaluate the association between the consumption of ultra‐processed foods (UPF) and the anthropometric profile of lactating women in situations of socioeconomic vulnerability. In this cross‐sectional study, we collected socioeconomic and health data, food consumption information, and anthropometric measurements of lactating women between 30 and 150 days postpartum. Food consumption was evaluated using a 24‐h dietary recall adapted to UPF through the Nova classification, and the anthropometric profile was assessed according to postpartum weight retention (kilograms (kg)), current body mass index (BMI) (kg/m^2^), and measurements of body perimeters (centimeters (cm)) and skinfolds (millimeters (mm)). Participants were grouped according to the proportion of dietary energy derived from UPF (tertile 1–2 vs. tertile 3). Adjusted linear regression models were employed to analyze associations. The study population consisted of 124 lactating women, most of had low income (102 (82.3%)) and lowest level of education (113 (91.1%)), and the average proportion of dietary energy from ultra‐processed foods (UPF) was 25% (95% confidence interval (95% CI) 0%–76%). We found a positive association between the proportion of dietary energy from UPF and postpartum weight retention (*β* = 3.75, 95% CI 1.40–6.10, *p* < 0.002). Our findings suggest that a greater proportion of UPF in the diet of lactating women is related to postpartum weight retention, which reinforces the need for actions aimed at reducing UPF consumption during lactation. Our results require confirmation from future, more rigorous studies.

## Graphical abstract

Our findings suggest that a higher proportion of ultra‐processed foods (UPF) in the diet of lactating women is associated with postpartum weight retention. This highlights the importance of implementing strategies to reduce UPF consumption during the lactation period.

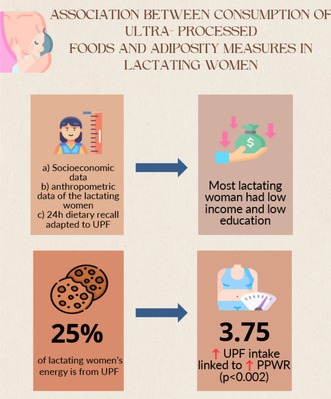

## 1 Introduction

According to data from the (World Health Organization (WHO) 2024), overweight is present in 44% of women, and of these, 18% have obesity. This multifactorial disease has several risk factors, such as postpartum weight retention (PPWR) (Kumari et al. 2022), which is influenced by gestational weight gain (Rong et al. 2015), pre‐pregnancy body mass index (BMI) (Hollis et al. 2017), parity (Hill et al. 2017), breastfeeding (Jiang et al. 2018), physical activity, use of contraceptives (Østbye et al. 2012), and maternal diet (Bazzazian et al. 2023).

PPWR has been demonstrated to serve as a reliable predictor of obesity and has been associated with an elevated risk of developing chronic diseases, including cardiovascular disease, diabetes, and certain types of cancer (Chescheir 2011; Galassi et al. 2006; Mamun et al. 2010). Additionally, PPWR has been associated with adverse outcomes in subsequent pregnancies, including preeclampsia, gestational diabetes mellitus, caesarean section, stillbirth, congenital anomalies, increased birth weight, and large‐for‐gestational‐age birth. These outcomes can have a significant impact on maternal and child health (Bogaerts et al. 2013; Ehrlich et al. 2011; McBain et al. 2016). This results in a substantial economic burden, manifesting in increased healthcare expenditures, diminished productivity, elevated rates of disability, and a reduction in the expected duration of a healthy, disability‐free life throughout the life course (The World Bank 2020).

In this context, the literature demonstrates that the quality of the maternal diet during lactation can significantly reduce body weight retention and the accumulation of maternal adiposity. However, a higher intake of foods that are high in energy, sodium, cholesterol, and trans fats, and low in fiber (profile of ultra‐processed foods (UPF)) may be related to greater PPWR (Leghi et al. 2021; Monteiro et al. 2019).

UPF are defined as industrialized foods that are primarily composed of substances extracted from the whole food. These substances undergo chemical modifications, the addition of nonculinary ingredients, and frequent alterations with the incorporation of food additives and sophisticated packaging (Monteiro et al. 2019).

The consumption of UPF has been associated with increased risks of weight gain and obesity in adults (Dicken and Batterham 2024). Additionally, pregnant women with high intakes of UPF were three times more likely to be obese than those with low intakes (Sartorelli et al. 2019). Furthermore, UPF consumption has been shown to be higher in low‐income and food‐insecure populations, and it increased during the coronavirus disease 2019 (COVID‐19) pandemic (Andrade et al. 2023; Coletro et al. 2023).

The association between ultra‐processed food (UPF) consumption during lactation and postpartum weight retention (PPWR) has not yet been well established. However, it is important to consider the potential influence of UPFs on weight retention in the postpartum period, given their high prevalence in the diets of breastfeeding women, often replacing unprocessed or minimally processed foods (Cummings et al. 2022; de Oliveira et al. 2022).

Given the rising prevalence of maternal overweight (World Health Organization 2024), the well‐established association between postpartum weight retention (PPWR) and the development of overweight in women (Kumari et al. 2022), and the documented impact of ultra‐processed food (UPF) consumption on overweight in adults and childhood obesity (Dicken and Batterham 2024; Neri et al. 2022), the lack of evidence regarding UPF intake during lactation highlights a gap in the literature. Therefore, this study aims to investigate this association among breastfeeding women of low socioeconomic status.

## 2 Methods

### 2.1 Selection and Description of Participants

This study was reported in accordance with the Strengthening the Reporting of Observational Studies in Epidemiology (STROBE) statement checklist. A cross‐sectional study involving lactating women was recruited to participate by convenience during their appointments for the Child Growth and Development Program at primary healthcare centers of Natal, Rio Grande do Norte (RN), Brazil. Due to the outbreak of the COVID‐19 pandemic, data collection for the study was intermittently suspended and conducted from July 2021 to August 2023. Furthermore, access to health monitoring for the child population, where data collection was performed, was also compromised (Fundação Maria Cecilia Souto Vidigal 2022). Convenience sampling was justified by the difficulty in reestablishing appointments for children and lactating women at health units across the country, which had limited services due to the pandemic that ended only in May 2022. To ensure participant randomness, data collection was conducted in nine health units located in four health districts of Natal.

We included all adult women (> 20 years) with no upper or lower age limits for maternal subjects, who were between 30 and 150 days postpartum, who were breastfeeding their child, either exclusively or partially (in cases where the infant is fed with milk of a different type to that derived from the mother's breast). We excluded those diagnosed with diseases and complications during pregnancy (diabetes mellitus, hypertensive syndromes, neoplasms, gastrointestinal and liver diseases, heart diseases, syphilis, and human immunodeficiency viruses (HIV) positive), smokers, premature births (< 37 gestational weeks), multiple pregnancies, and newborns presented any malformation.

The postpartum interval (30–150 days) is supported by the relatively stable composition of breast milk and the recommendation to assess weight changes only after 6 weeks postpartum. Before this period, the body undergoes a phase of fluid and electrolyte rebalancing, during which the excess extracellular and extravascular water accumulated during pregnancy is reduced, and blood volume gradually returns to prepregnancy levels (Institute of Medicine 1991; Kumari et al. 2022).

### 2.2 Sample Calculation

The definition of this study's population was based on the sample analysis calculation considering a difference between proportions of UPF consumption in the breastfeeding population of another study conducted in the same region (lowest tertile of UPF 0%–8.6% and highest tertile 19.6%–53.9%) (Amorim et al. 2021). A 95% confidence interval was adopted, with 80% power (Lwanga et al. 1991). Thus, a sample of 119 women was determined; however, 124 participants were included in the study since 15% of losses were accounted for in the initial sample.

### 2.3 Ethical Aspects

The project was submitted to and approved by the Ethics and Research Committee of the Federal University of Rio Grande do Norte (UFRN) (CAAE 29928420.7.0000.5292), under code 4.199.673.

Prior to data collection, the research objectives and procedures were explained to the lactating women in language accessible to their educational level. Subsequently, the document was thoroughly reviewed to ensure that all pertinent information had been thoroughly recorded. Following this examination, the document was then presented to the individuals for their signature. The Informed Consent Form is a document that must be completed and signed by all participants prior to the initiation of any procedure. Subsequently, each participant was given a copy of the Informed Consent Form, while the remaining form was retained by the research team.

### 2.4 Data Collection and Measurements

**FIGURE 1:**
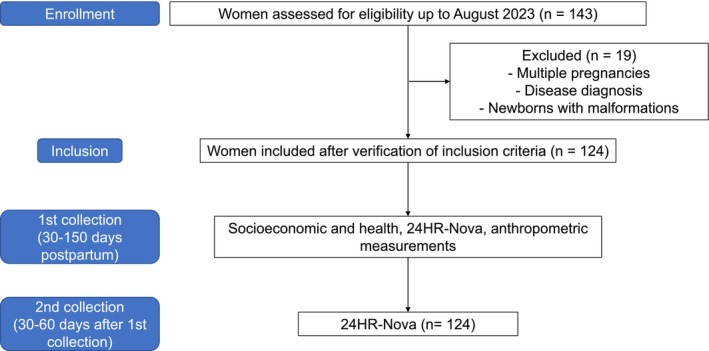
Flowchart of data collection. Rio Grande do Norte, Brazil (2021–2023). *Source:* Created by the authors (2024).

Data collection occurred in two stages. Firstly, a semi‐structured face‐to‐face interview was conducted with the lactating women through an electronic form that included three dimensions: (a) socioeconomic data, (b) anthropometric data of the lactating woman, (c) 24‐h dietary recall adapted to UPF (24HR‐Nova) (NUPENS/USP—Núcleo de Pesquisas Epidemiológicas em Nutrição e Saúde da Universidade de São Paulo 2013). After 30–60 days of the first data collection, a new 24HR‐Nova was applied to the lactating women in person at the health centers at their homes or remotely by phone (Figure 1). Consumption showed no significant difference between the two 24HR‐Nova (data not shown), likely due to the nature of UPFs, which are primarily composed of formulations of ingredients intended for industrial use. Unlike minimally processed foods, they are not affected by seasonal availability (Institute of Medicine 1991; Kumari et al. 2022; Monteiro et al. 2019).

### 2.5 Consumption of UPF

Maternal food intake was assessed using two 24HR‐Nova obtained through interviews in‐person or remotely according to the multiple‐pass method and supported by the GloboDiet photographic manual (Crispim 2017), on nonconsecutive days (no weekends) to capture dietary variability by dietitians and previously trained Nutrition undergraduate students. The remote collection was employed when in‐person interaction was not feasible. The data collection process involved the use of video calls for participants who were unable to be present at the primary healthcare service.

Usual dietary intake from 24HR‐Nova was calculated by Multiple Source Method (MSM) to reduce the effect of intrapersonal variability. The MSM's primary objective is to estimate usual intake distributions of nutrients and food intake, including episodically consumed foods, and it can accommodate short‐term measurements, such as 24‐h protocols. The MSM may also be applied provided that data collection occurs on randomly selected days (Harttig et al. 2011). A pilot study was conducted to assess the performance of the research instruments and the data collection protocol.

The Virtual Nutri Plus Software environment was used to create a food database based on the Brazilian Food Composition Table (TBCA) (NEPA‐UNICAMP 2011) and food equivalents from the Nutrient Database for Standard Reference of the United States Department of Agriculture (USDA) (USDA Food and Nutrient Database for Dietary Studies 2004). Foods were classified according to the extent of industrial processing using the Nova classification system (Monteiro et al. 2019).

Nova includes 4 food groups. Group 1 consists of unprocessed or minimally processed foods, such as fresh, dried, or frozen fruits and vegetables, grains, legumes, meat, fish, and milk, which have undergone minimal processing like grinding, roasting, pasteurization, or freezing. Group 2 includes processed culinary ingredients, such as table sugar, oils, salt, and other substances that have been extracted, pressed, or centrifuged from Group 1 foods or from nature, and are used to make culinary preparations; to identify Group 2, the recipes were disaggregated. Group 3 includes processed foods, which are made using unprocessed or minimally processed foods, and ingredients from Group 2 are used to extend the shelf life of foods and enhance their palatability. Examples of foods in Group 3 include canned fruits, artisanal bread and cheese, and salted, smoked, or cured meat or fish. Group 4 consists of UPFs, which are formulations of several ingredients from Group 2 with food additives not used in home preparations, such as flavors, colors, sweeteners, emulsifiers, and other substances used to mask undesirable qualities of the final product or mimic the sensory qualities of culinary preparations from Group 1 (Monteiro et al. 2019). Then, the relative energy proportion of the UPF in relation to the total energy consumption of the diet was obtained, and the lactating women were grouped according to the tertiles of UPF consumption.

Food items were sorted into food subgroups within the four Nova food groups. Thus, the UPF were grouped by similarity, based on the study by Louzada et al. (2023), with adaptations that included some food groups with higher consumption of certain foods by the participants. The dietary share of UPF (and subgroups within them) to the total energy intake was calculated.

### 2.6 Anthropometric Profile and Anthropometric Measures

Anthropometric measures were used to evaluate the anthropometric profile of the lactating women. The study team collected weight and height measurements using techniques proposed by Lohman et al. (1998) and these were gathered by dietitians and previously trained Nutrition undergraduate students. Weight was obtained using a portable electronic scale, and height was measured in duplicate with a portable anthropometer during the interview. Participants were classified into three BMI categories: < 18.5 kg/m^2^ (underweight), 18.5–24.9 kg/m^2^ (normal weight), and ≥ 25 kg/m^2^ (World Health Organization 1995; World Heath Organization 2000).

PPWR (kg) was calculated as the difference between the current postpartum weight and self‐reported weight before pregnancy. High PPWR was higher than or equal to 4.0 kg in accordance with Kumari et al. (2022).

Other anthropometric measurements (neck, hip, and thigh circumference) were also collected by dietitians and previously trained Nutrition undergraduate students using inelastic fiberglass anthropometric tape. Triceps and subscapular skinfold measurements were taken using a Lange adipometer. These skinfold measurements were used for the sum of skinfolds in the analysis. The measurements were performed according to the technique proposed by Lohman et al. (1998) and described in millimeters (mm) and centimeters (cm).

### 2.7 Covariates

We also gathered information through the electronic questionnaire, which was used as control covariates for the statistical analysis, such as age and maternal education, date of delivery, type, parity, and days postpartum, gestational age at birth and prepregnancy BMI (obtained from medical records, when such records were available, in cases where records were not available, the researchers conducted interviews with lactating women and recorded the data in the electronic questionnaire), number of household residents, family income, and income changes caused by the COVID‐19 pandemic. Maternal education was classified as lowest level of education and highest level of education (Instituto Brasileiro de Geografia e Estatística 2023).

To assess per capita income, we divided the reported family income by the number of household residents and compared it with the World Bank goal (World Bank Group—International Development, Poverty, and Sustainability, de 2022), which classifies poverty as a situation where per capita family income is less than half the minimum wage, which is less than US$116.81.

Information on anthropometric data before and during pregnancy (prepregnancy BMI was calculated by body weight prepregnancy (kg) divided by height squared (m^2^)), classified as underweight, normal weight, overweight, and gestational weight gain, categorized as below recommended, within recommended, and above recommended, was obtained from the prenatal records and analyzed according to the classification of the Australian Department of Health (Ministério da Saúde 2022).

### 2.8 Statistical Analysis

For the statistical analyses, the participants were divided into two groups: those in the highest tertile of UPF consumption, which refers to individuals in the top third of energy contribution from UPF relative to the total calorie intake of the diet (T3, ≥ tertile 3), and those below that (T1‐2, tertiles 1 and 2), to assess the differences and relationships between the highest energy share of UPF consumption and the anthropometric profile.

Model analyses using multiple linear regression were conducted to evaluate the association between T3 (outcome variable) and anthropometric measurements of lactating women. These analyses were adjusted for control covariates including income (low income | no low income), education (lowest level of education | highest level of education), categorized prepregnancy BMI, categorized gestational weight gain, exclusive breastfeeding (yes | no), number of children, and the number of days postpartum. The covariates were introduced into the models using the stepwise method for estimating the beta values and respective 95% confidence intervals, along with the *p* value. In the end, all regression models were presented (raw and adjusted models). Statistical significance was set at *p* < 0.05 two‐sided. Data analysis was performed using the Statistical Package for the Social Sciences (IBM SPSS) version 20.0.1.1.

## 3 Results

Overall, 124 lactating women were included in the analyses. The sociodemographic and health characteristics of the population are detailed in Table 1. The studied population was characterized by women with a mean age of 28 (7) years, living below the poverty line (82.3%) and with low levels of education, showing a statistical difference in education levels according to the tertiles of UPF contribution. Women in the group with the lowest proportion of UPF in their diet (tertile 1 and 2) had a higher level of education (*p* = 0.015). Furthermore, more than half of the participants were classified as prepregnancy overweight and experienced high gestational weight gain, with 71.8% of the women practicing exclusive breastfeeding.

**TABLE 1:** Sociodemographic characteristics of lactating women grouped by tertiles of energy contribution of ultra‐processed foods in the total energy consumed, Rio Grande do Norte, Brazil (2021–2023).

Sociodemographic and health variables	Total sample	Percentage of energy contribution of ultra‐processed foods		*p*
	*n* (124)	Tertile 1–2 (0%–29.53%)	Tertile 3 (> 29.54%)	
		*n* (84)	*n* (40)	
Age in years, mean (SD)	28 (7)	28 (7)	27 (6)	0.174^b^
Marital status				
Single, *n* (%)	40 (32.3)	26 (31.3)	14 (34.1)	0.752
Married/de facto relationship, *n* (%)	84 (67.7)	57 (68.7)	27 (65.9%)	
Education Level				
Lower level of education, *n* (%)	113 (91.1)	72 (86.7)	41 (100.0)	**0.015**
Highest level of education, *n* (%)	**11 (8.9)**	**11 (13.3%)**	**0 (0.0)**	
Number of people in the household, mean (SD)	4 (3–4)	4 (3–6)	4 (3–6)	0.451^b^
Household income per capita, *n* (%)^a^				
< USD 116.81	102 (82.3)	67 (80.7)	35 (85.4)	0.524
≥ USD 116.81	22 (17.7)	16 (19.3)	6 (14.6)	
Change in income during COVID‐19, *n* (%)				
No	67 (54.9)	42 (51.2)	25 (62.5)	0.240
Yes	55 (45.1)	40 (48.8)	15 (37.5)	
Birth type				
Vaginal birth, *n* (%)	62 (50)	42 (50.6)	20 (48.8)	0.849
Caesarean section, *n* (%)	62 (50)	41 (49.4)	21 (51.2)	
Number of postpartum days, median (Q1–Q3)	68 (42–104)	64 (40–96)	83 (58–132)	0.059^c^
Parity, median (Q1–Q3)	2 (1–2.25)	2.0 (1.0–2.0)	2.0 (1.0–3.0)	0.474^c^
Interbirth interval, median (Q1–Q3)	2.9 (0.0–7.0)	4.0 (0.0–8.0)	2.0 (0.0–6.0)	0.089^c^
Exclusive breastfeeding, *n* (%)				
Yes	89 (71.8)	58 (69.9)	31 (75.6)	0.505
No	35 (28.2)	25 (30.1)	10 (24.4)	

*Note:* Bold values are significant (*p* < 0.05).

^a^ Poverty line established based on the World Bank (< 1/2 minimum wage = BRL 660.00).

^b^ *T*‐Student test.

^c^ Mann–Whitney test.

**FIGURE 2:**
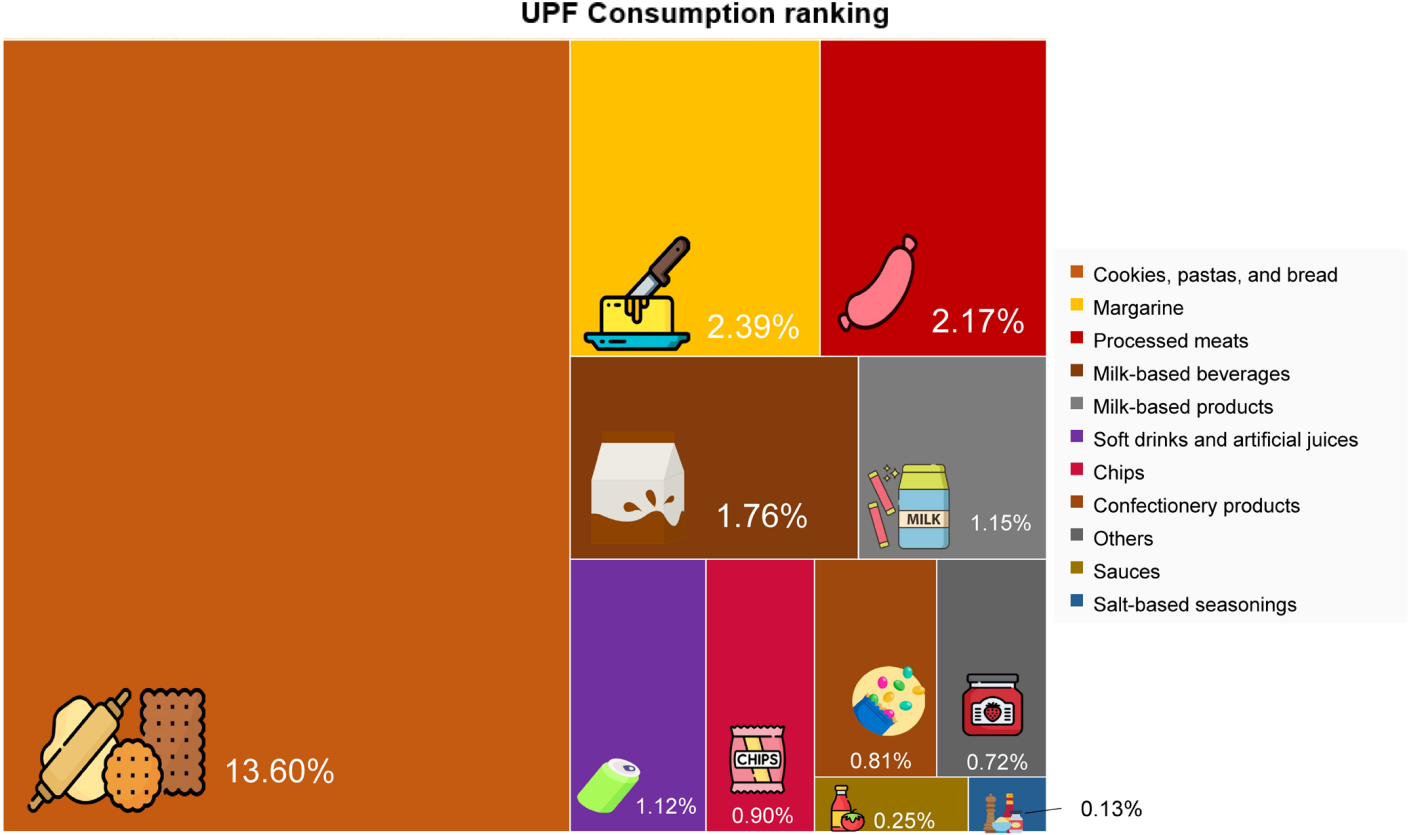
Main UPF found according to energy proportion in the diet of lactating women (*N* = 124). Rio Grande do Norte, Brazil (2021–2023). Percentage derived from the 25% of UPF consumed daily in the diet of lactating women. *Source:* Created by the authors (2024)

The average caloric intake of lactating women was 2740 Kcal (1068–6076 Kcal), with 51% of energy intake sourced from unprocessed and minimally processed foods and 25% from ultra‐processed foods (UPF) (Table 2), primarily from biscuits, pasta, and bread (13.60%), margarine (2.39%), and processed meats (2.17%) (Figure 2). When analyzing the anthropometric profile during lactation, overweight according to BMI was observed in 65.5%, and 40.9% of these individuals presented with PPWR greater than 4 kg, with higher values of weight retention in women who had a greater caloric proportion of UPF in their diet (3.9 kg in T3 vs. 2.9 kg in T1‐2, *p* = 0.027). Regarding the other anthropometric measurements, there appears to be a trend towards higher values of thigh and hip circumference in the highest tertile of UPF contribution, though there were no statistically significant differences between the groups (Table 3).

**TABLE 2:** Average relative proportion of unprocessed or minimally processed foods, processed culinary ingredients, processed foods, and ultra‐processed foods in the total energy consumed by lactating women (*n* = 124), Rio Grande do Norte, Brazil (2021–2023).

Food group according to its processing level^a^	Dietary variables	
	Energy—Kcal/day	% of total energy
	Mean (CI)	Mean (CI)
Unprocessed and minimally processed foods	1401 (345–3110)	51 (17–94)
Culinary ingredients	329 (19–1327)	12 (1–35)
Processed foods	319 (0–1208)	12 (0–45)
Ultra‐processed foods	690 (0–3319)	25 (0–76)
Total	2740 (1068–6076)	100

^a^ Monteiro et al. (2019).

*Source:* Created by the authors (2024).

**TABLE 3:** Anthropometric characteristics of lactating women grouped by tertiles of energy contribution of ultra‐processed foods in the total energy consumed, Rio Grande do Norte, Brazil (2021–2023).

Anthropometric characteristics	Total sample	Percentage of energy contribution of ultra‐processed foods		*p*
	*n* (124)	Tertile 1–2 (0%–29.53%)	Tertile 3 (> 29.54%)	
		*n* (84)	*n* (40)	
Pre‐pregnancy BMI (kg/m^2^), *n* (%)^c^				
Underweight	6 (5.3)	4 (5.3)	2 (5.3)	0.916
Normal weight	42 (36.8)	29 (38.2)	13 (34.2)	
Overweight	66 (57.9)	43 (56.6)	23 (60.5)	
Gestational weight gain (kg), *n* (%)^c^				
Below recommended	15 (14.2)	12 (17.1)	3 (8.3)	0.277
Within recommended	22 (20.8%)	16 (22.9)	6 (16.7)	
Above recommended	69 (65.1)	42 (60.0)	27 (75.0)	
Current BMI (kg/m^2^), *n* (%)^d^				
Underweight	3 (2.7)	3 (3.8)	0 (0)	0.455
Normal weight	35 (31.3)	23 (29.5)	12 (35.3)	
Overweight	74 (66.0)	53 (66.7)	22 (64.7)	
PPWR (kg), median (Q1–Q3)^e^	3.0 (−0.3–5.0)	2.90 (−0.9–4.5)	3.9 (0.0–10.4)	**0.027** ^a^
Thigh circumference (cm), mean (SD)	54.0 (49.8–57.8)	53.0 (49.7–57.0)	55.0 (50.0–64.8)	0.076^a^
Neck circumference (cm), median (Q1–Q3)	33.5 (32.0–36.0)	33.5 (32.0–35.0)	34.0 (32.1–36.0)	0.414^a^
Hip circumference (cm), mean (SD)	104.16 (12.51)	102.60 (13.01)	107.40 (10.96)	0.099^b^
Sum of skinfolds (mm), mean (SD)^f^	45.41 (16.30)	43.75 (16.03)	48.71 (16.44)	0.141^b^

*Note:* Bold values are significant (*p* < 0.05).

^a^ Mann–Whitney test.

^b^ *T*‐Student test.

^c^ Ministério da Saúde (2022).

^d^ World Heath Organization (2000).

^e^ Kumari et al. (2022).

^f^ Skinfolds = triceps skinfold + subscapular skinfold.

Multiple linear regression models indicated that a higher caloric proportion of UPF (T3) in the diet was positively associated with PPWR (*β* = 3.75, 95% CI 1.40–6.10, *p* < 0.002) (Table 4). The other measurements did not show a significant association with UPF consumption but were positively associated with prepregnancy BMI, which emerged as the variable with greater explanatory power.

**TABLE 4:** Association of the highest relative proportion of UPF in the diet consumed and indicators of adiposity in lactating women. Rio Grande do Norte, Brazil (2021–2023).

Adiposity measures	Highest tertile (T3) of caloric contribution of UPF in the maternal diet		
	*β*	95% CI	*p*
PPWR (kg)			
Model 1			
T3% UPF	3.62	1.14 to 6.11	0.005
Model 2			
T3% UPF	3.75	1.40 to 6.10	0.002
Prepregnancy BMI (classification)^a^	−3.32	−5.13 to −1.51	< 0.001
Model 3			
T3% UPF	3.48	1.34 to 5.62	0.002
Prepregnancy BMI (classification)^a^	−3.80	−5.46 to −2.15	< 0.001
Gestational weight gain (classification)^a^	3.23	1.88 to 4.59	< 0.001
Current BMI (kg/m^2^)			
Model 1			
Prepregnancy BMI (classification)^a^	6.61	5.05 to 8.17	< 0.001
Model 2			
Prepregnancy BMI (classification)^a^	6.37	4.84 to 7.90	< 0.001
Gestational weight gain (classification)^a^	1.59	0.34 to 2.84	0.013
Thigh circumference (cm)			
Model 1			
Prepregnancy BMI (classification)^a^	6.78	4.31 to 9.25	< 0.001
Model 2			
Prepregnancy BMI (classification)^a^	6.49	4.06 to 8.91	< 0.001
Gestational weight gain (classification)^a^	2.39	0.41 to 4.37	0.018
Model 3			
Prepregnancy BMI (classification)^a^	6.85	4.54 to 9.16	< 0.001
Gestational weight gain (classification)^a^	2.57	0.69 to 4.45	0.008
Number of births	−1.75	−2.74 to −0.76	0.001
Neck circumference (cm)			
Model 1			
Prepregnancy BMI (classification)^a^	2.65	1.44 to 3.86	< 0.001
Hip circumference (cm)			
Model 1			
Prepregnancy BMI (classification)^a^	12.37	7.86 to 16.88	< 0.001
Model 2			
Prepregnancy BMI (classification)^a^	11.14	6.75 to 15.53	< 0.001
Gestational weight gain (classification)^a^	4.62	1.23 to 8.01	0.008
Sum of skinfolds (mm)^b^			
Model 1			
Prepregnancy BMI (classification)^a^	16.24	11.67 to 20.81	< 0.001

^a^ Ministério da Saúde (2022).

^b^ Skinfolds = triceps skinfold + subscapular skinfold.

## 4 Discussion

This study evaluated the association between the consumption of UPF and anthropometric measurements and indicators related to adiposity in lactating women in situations of socioeconomic vulnerability. One of our findings that was not available in the literature is that a higher contribution of UPF in the diet was associated with greater PPWR.

The consumption pattern of UPF varies within the population. Women with a higher level of education consumed less UPF, as demonstrated by this study. This finding is consistent with the results reported by Coletro et al., highlighting that in Brazil, this consumption is directly associated with food insecurity and closely linked to socioeconomic factors (Coletro et al. 2023).

A survey conducted with the Brazilian population during the COVID‐19 pandemic revealed that between 2019 and 2021, and between 2020 and 2021, there was a significant decrease in the consumption of cereals, vegetables, fruits, and industrial fruit juices. Conversely, there was an increase in the consumption of soft drinks, sweet or sandwich biscuits, packaged cakes/muffins, processed meats, margarine, industrial sauces, and ready‐to‐eat meals, with the decline in family income due to the pandemic being one of the main reasons for these changes in consumption (Andrade et al. 2023; Coletro et al. 2023).

Moreover, the consumption of UPF during lactation varies (de Sousa et al. 2025). In American postpartum women, according to The Pregnancy Eating Attributes Study (PEAS), the caloric proportion of UPF was 50.6% (Nansel et al. 2022). In this study, the contribution of UPF represented a quarter of the women's diet, which was higher than the consumption found in women during lactation in the same region during the pre‐pandemic period (Amorim et al. 2021). Although this consumption has increased, it remains relatively low in comparison to other countries (Nansel et al. 2022; Rauber et al. 2021). This may be attributed to the prominence of traditional Brazilian foods, such as beans and rice, which have higher average daily consumption per capita: 142.2 g/day and 131.4 g/day, respectively (Instituto Brasileiro de Geografia e Estatística 2020).

The average caloric intake observed in this population is notably higher than those in other studies (Amorim et al. 2021; Ferreira et al. 2018; Wang et al. 2021). However, an analysis of the guidelines reveals a predominant tendency to increase energy intake for the purpose of lactation, with the recommended daily caloric intake of approximately 500 kcal per day. This recommendation does not establish a mean daily caloric intake (Ministério da Saúde 2009; Trumbo et al. 2002; United Nations University et al. 2005).

The increase in UPF consumption in the population in general, and particularly in lactating women, is an important aggravating factor for health, in view of the latest observations in the literature that show the association of increased consumption with a higher risk of developing cardiovascular disease, hypertension (Pant et al. 2023), risk of all‐cause mortality, cerebrovascular disease, depression (Pagliai et al. 2021), type 2 diabetes mellitus (Chen et al. 2023), and obesity (Rauber et al. 2021).

As in this study, Louzada et al. (2023) observed the presence of margarine (2.68%), biscuits (2.41%), and bread (1.94%) as the most consumed ultra‐processed products in the Brazilian population, with higher consumption among females, those with lower education, and individuals on low incomes PPWR has shown an average variation between women of 0.5 and 3 kg, with up to 20% of women retaining more than 4 kg 1 year after delivery, as indicated by Siega‐Riz et al. (2009).

The present study has shown a statistically significant difference of ~1 kg in weight retention between UPF consumption. Although small, it is important to highlight that postpartum weight retention at this stage of lactation may be a determining factor for overweight in women. These participants already present risk factors associated with excess weight, such as low socioeconomic status, high prepregnancy BMI, and excessive gestational weight gain. When combined with reduced energy expenditure due to decreased breastfeeding or weaning, these factors may further increase the risk of overweight (Kossou et al. 2023).

Our study found a positive association between higher consumption of ultra‐processed foods (UPF) during lactation and greater PPWR. To date, there has been no research observing the influence of UPF consumption during the lactation phase on PPWR, which may be due to its association with a lower overall diet quality in these women, often characterized by high energy density, sodium, simple sugars, and saturated and trans fats, as well as the use of additives and low levels of protein, fiber, vitamins, and minerals (Monteiro et al. 2019).

The absence of a relationship between UPF and BMI and skinfolds in this population may be attributed to certain experiences during this biological cycle, such as breastfeeding and the presence of hormones characteristic of the lactogenesis process, such as prolactin, which help mobilize the weight gained during pregnancy (Lopez‐Vicchi et al. 2020; McClure et al. 2012).

Prepregnancy BMI was the strongest predictive variable for the other measures of adiposity, as identified by Ahmadibeni et al. (2023) who observed a decrease in the reduction of hip circumference and PPWR in women with a higher prepregnancy BMI. In this study, we do not have an evaluation of UPF consumption before and during pregnancy, but the literature already highlights the influence that UPF has on overweight (Liu et al. 2023), indicating the crucial role of prepregnancy BMI in the subsequent distribution of adiposity across different measures in the postpartum period (Lopez‐Vicchi et al. 2020).

Campbell et al. (2016) demonstrated that women who are overweight or have obesity are more likely to experience excessive gestational weight gain and PPWR, as well as an increased risk of health outcomes such as gestational hypertension, diabetes, preterm delivery, macrosomia, and caesarean section (Chen et al. 2020). This is also linked to a higher risk of PPWR and long‐term obesity (Sawangkum and Louis 2020). The gestational weight gain observed in this population aligns with findings in the literature, as cited by Gilmore et al. (2015), who state that approximately half of all women exceed the recommendations for gestational weight gain.

This study has some limitations: (1) the short postpartum period during which the lactating women were evaluated; although there is no indication in the literature of the optimal period for evaluation, it was conducted within the minimum timeframe to observe changes in weight retention (after 6 weeks postpartum) (Kumari et al. 2022); (2) obtaining a single measurement of circumferences, perimeters, and skinfolds, which were assessed only at one postpartum moment, making it impossible to track their variation over time; (3) the evaluation of food consumption is constrained by limitations such as the utilization of only two 24HR‐Nova on weekdays, in conjunction with the lactation stage, which may influence the interviewee's memory, fatigue levels, and awareness concerning the foods employed in the recipes. This is due to the necessity of estimating the list of ingredients for certain culinary dishes.

However, the strengths of this research are also worth highlighting, such as the use of 24HR‐Nova, a specific tool for evaluating food consumption based on the Nova classification. This involved using a standardized statistical code and breaking down the dishes into their underlying ingredients to ensure the accurate application of the classification system. Additionally, the recalls were subjected to stages of critical consumption analysis, conducted by researchers who were previously trained to collect this information and perform anthropometric measurements. Also noteworthy is the large proportion of women engaged in exclusive breastfeeding (more than 70%) and partial breastfeeding, which provides reliable information on the impact of UPF consumption even in the context of breastfeeding.

Furthermore, this was the first study to assess the relationship between UPF consumption and the anthropometric profile during lactation, introducing innovation to the current understanding of the impact of UPF consumption on maternal and child health, as well as factors associated with PPWR and adiposity in women. These data are important for expanding actions to protect maternal and child health, such as emphasizing the importance of guiding the consumption of unprocessed and minimally processed foods during this phase. It is worth mentioning that this research was conducted during the period of the Covid‐19 pandemic, and it was a time when there was a general decrease in the number of records of consultations used to assess the growth and development of children, and it involved a socioeconomically vulnerable population that is similar to most of the Brazilian population. Future research involving lactating women over a longer postpartum period would be beneficial to elucidate the relationship more comprehensively between UPF consumption and body composition at the end of lactation and beyond this phase.

In conclusion, the findings of this study indicate that a higher proportion of UPFs in the diet is associated with greater postpartum weight retention (PPWR), while prepregnancy BMI was a significant predictor of adiposity measures. These results highlight the importance of considering the degree of food processing as a strategic component in efforts to prevent and address obesity within the context of women's health.

## Author Contributions

**Lara Virginia Pessoa de Lima:** formal analysis (equal), investigation (equal), writing – original draft (equal). **Lígia Rejane Siqueira Garcia:** conceptualization (supporting), writing – review and editing (supporting). **Juliana Morais de Sousa:** investigation (equal), writing – original draft (supporting). **Priscila Gomes de Oliveira:** investigation (equal), writing – original draft (supporting). **Nicolie Mattenhauer de Oliveira:** investigation (supporting). **Juliana Fernandes dos Santos Dametto:** conceptualization (supporting), writing – review and editing (supporting). **Danielle Soares Bezerra:** conceptualization (supporting), writing – review and editing (supporting). **Karla Danielly da Silva Ribeiro:** conceptualization (lead), formal analysis (lead), investigation (lead), project administration (lead), supervision (equal), writing – original draft (lead), writing – review and editing (lead).

## Ethics Statement

Approved by the Ethics and Research Committee of the Federal University of Rio Grande do Norte (UFRN) (CAAE 29928420.7.0000.5292), under code 4.199.673.

## Conflicts of Interest

The authors declare no conflicts of interest.

## Data Availability

The data that support the findings of this study are available from the corresponding author upon reasonable request. The data are not publicly available due to privacy or ethical restrictions.
